# Supplementary material for: Heading representations in primates are compressed by saccades
Source: Nat Commun. 2017 Oct 13;8:920. doi: 10.1038/s41467-017-01021-5 (PMC5640607; doi:10.1038/s41467-017-01021-5)
Supplement: Supplementary file 1 — Supplementary Information [file 41467_2017_1021_MOESM1_ESM.pdf]

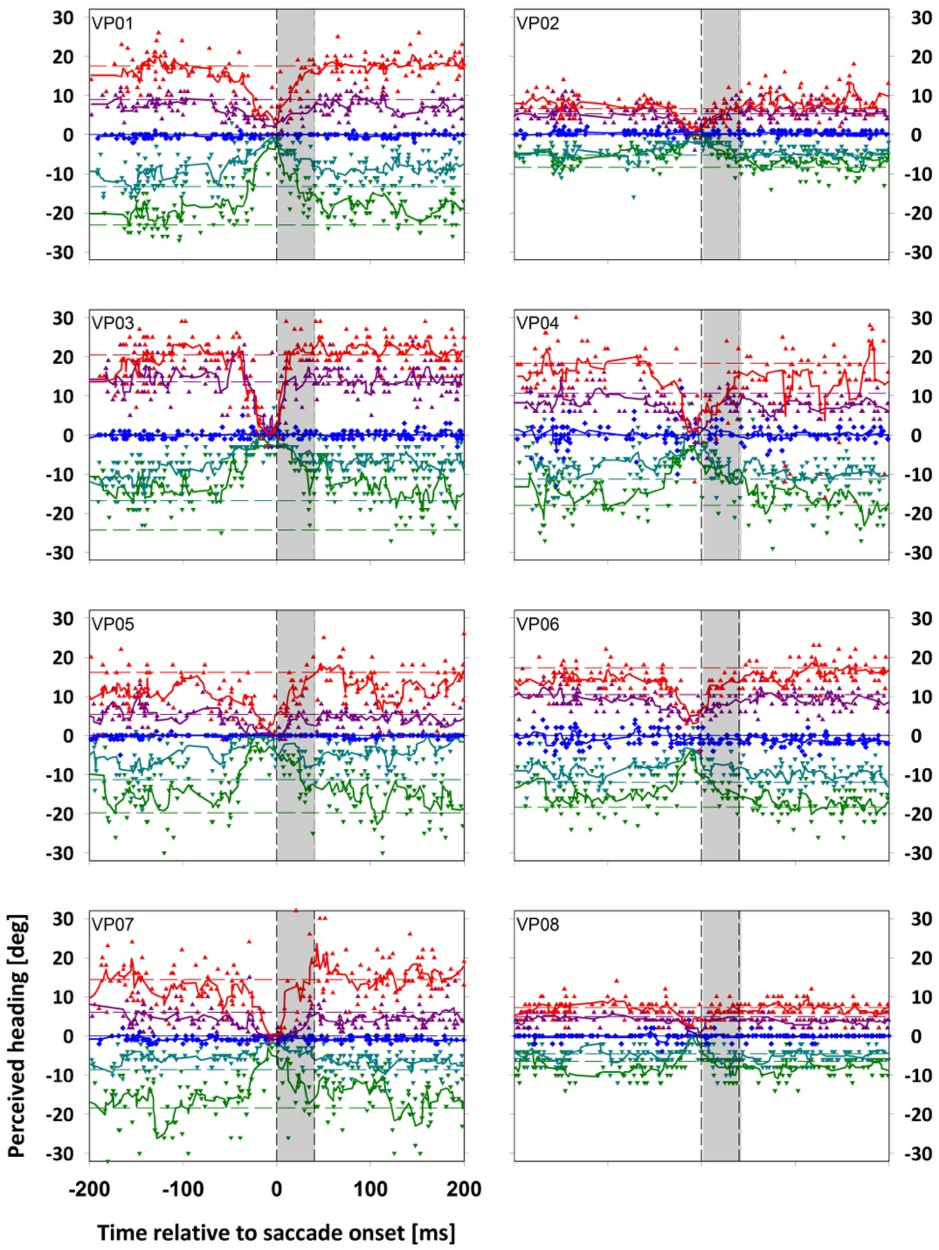

Supplementary Figure 1: Perisaccadic perception of heading. Behavioral responses from all subjects (VP01 – VP08). Symbols represent data from single trials: upward pointing triangles for heading to the right (magenta: +15°, red: +30°), downward pointing triangles for heading to the left (dark cyan: -15°, green: -30°), and circles for heading straight-ahead (blue, 0°). Each data point indicates the onset time of the 40 ms self-motion sequence (x-value) relative to saccade onset and the perceived heading (y-value). Solid colored lines represent running means of five consecutive samples each, assigned to the central sample value. Dashed horizontal lines show the performance for the same subjects during continuous fixation, as measured in different blocks of trials. For each subject, perceived heading during fixation trials was slightly biased towards fixation, as indicated by the dashed, colored horizontal lines. For subjects 2 (VP02) and 8 (VP08), this bias towards fixation was strongest.

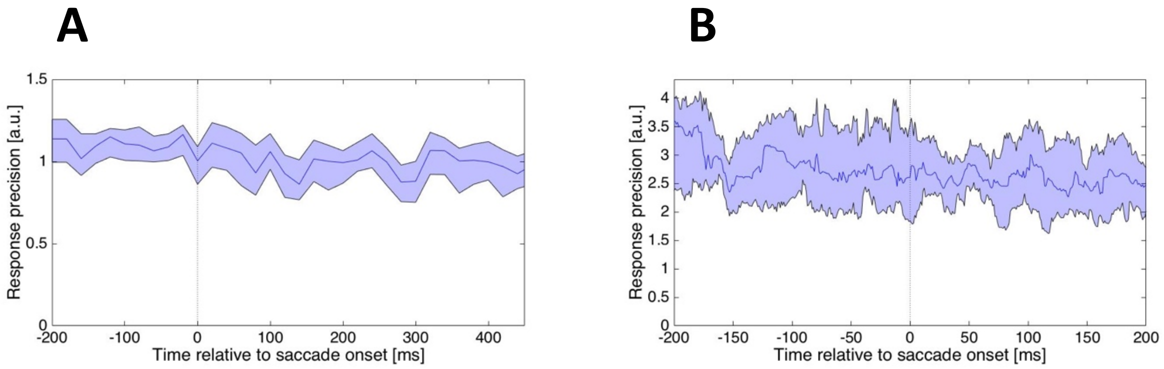

Supplementary Figure 2: Precision of decoded and perceived heading relative to saccade onset. (A) Precision of the decoded heading. We computed precision as the standard deviation of three consecutive samples. The data curve shows the mean precision across all three heading directions and the 95% confidence interval as determined from bootstrapping. (B) We determined the precision of the subject's responses by computing the standard deviation of nine consecutive behavioral responses. The average temporal interval between two samples was 2.5ms. The data curve (blue line) shows the time-resolved average precision across all subjects and all heading directions and the bootstrapped 95% confidence interval. In both cases, decoding and heading perception, the precision of the responses did not increase in the temporal vicinity of the saccade, indicated by the dashed vertical line.

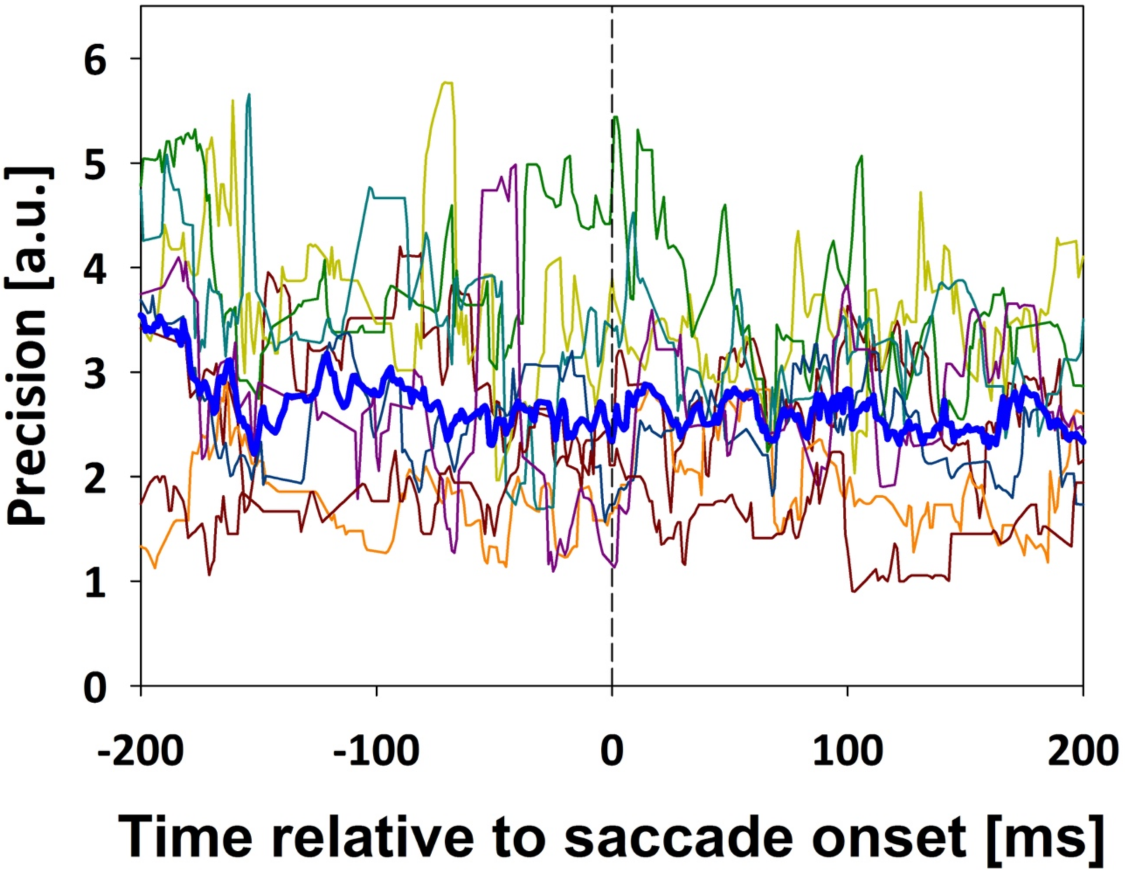

Supplementary Figure 3: Precision of heading perception at the single subject level. We computed precision as the standard deviation of nine consecutive samples of perceived heading (see Methods and **Fehler! Verweisquelle konnte nicht gefunden werden.** for further information). Each thin colored line represents data from a single subject. The thick blue line depicts the average time course of this precision measure across all subjects. There was no systematic perisaccadic modulation of the precision of heading perception.

	Area MST number / %	Area VIP number / %
Monkey R	5 of 9 / 56%	
Monkey H	44 of 55 / 80%	10 of 36 / 28%
Monkey C		12 of 19 / 63%

Supplementary Table 1: Number and proportion of neurons contributing to the decoding of heading. We applied the decoder to the continuously recorded discharge of each neuron that showed a statistically significant tuning for heading (see Methods for details). In total, these were 71/119 = 60% of the neurons. In detail, for area MST it were 5/9 (56%) neurons from monkey R and 44/55 (80%) neurons from monkey H. For area VIP, it were 10/36 (28%) neurons from monkey H and 12/19 (63%) neurons from monkey C.
